# Supplementary material for: OCP–FRP protein complex topologies suggest a mechanism for controlling high light tolerance in cyanobacteria
Source: Nat Commun. 2018 Sep 24;9:3869. doi: 10.1038/s41467-018-06195-0 (PMC6155142; doi:10.1038/s41467-018-06195-0)
Supplement: Supplementary file 1 — Supplementary Information [file 41467_2018_6195_MOESM1_ESM.pdf]

Supplementary information

OCP–FRP protein complex topologies suggest a mechanism for controlling high light tolerance in cyanobacteria

Sluchanko et al.

Supplementary Table 1 Structural parameters for FRP-L49E determined by SAXS.

Parameter	FRP-L49E	
Calculated mass	14.1 kDa	
Number of residues	124 (with His-tag)	
Protein concentration	1.2 mg ml^{-1}	4.0 mg ml^{-1}
R_g (Guinier)	2.18 ± 0.06 nm	2.56 ± 0.01 nm
R_g (reciprocal)	2.37 ± 0.01 nm	2.80 ± 0.02 nm
D_{max}	10 nm	12 nm
Porod volume	31 nm^3	39.7 nm^3
M_w (Porod)	19.2 kDa	24.8 kDa
M_w (MoW)	16.5 kDa	21.9 kDa
M_w (V_c)	17.9 kDa	24.1 kDa
Kratky plot	bell-shaped (folded)	bell-shaped (folded)

All SAXS-derived parameters were calculated using the ATSAS 2.8 software package[1].

Supplementary Table 2 Primers used in the study

Name	Sequence
L49E-forward	5’-GATTGATGATGAGTGGAAACTGCATG-3’
L33C/I43C reverse	5’-CAATCTGGGTACACTGGCTTGCCTGTGCCTGAACGGTTGCACACAGACCATCCAGTTC-3’
T5 forward	5’-GTGAGCGGATAACAATTTCACAC-3’
pQE reverse	5’-CATTACTGGATCTATCAACAGGAG-3’
F76C forward	5‘-GTGATCATTTGTGTTTTTGCAC-3‘
F76C reverse	5‘-GCTCTGACGATCATCATATTTG-3‘
K102C forward	5’-TAAACAGAGCTGCATCAAAGCACTGGC-3’
K102C reverse	5’-TCTGCTGCCAGAAAGGTC-3’
F299C forward	5‘-GGCAAAATCTGTTTTGTTGCC-3‘
F299C reverse	5‘-TTCCGGATTCAGCAGAAAAC-3’

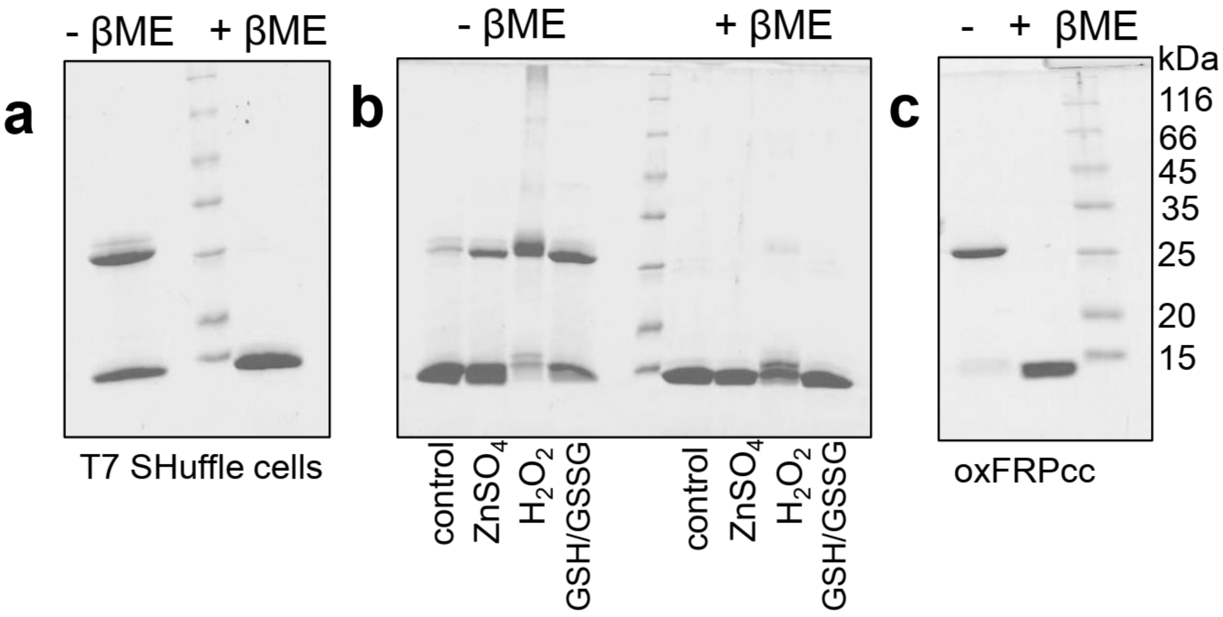

Supplementary Fig. 1 Formation of the disulfide trapped FRPcc mutant by oxidation. **a** Expression in T7 SHuffle cells and protein purification in the absence of reducing agents resulted in incomplete crosslinking of FRP dimers, showing also batch-to-batch variation. The dimers formed could be disrupted by addition of 20 mM βME and sample boiling prior to SDS-PAGE. **b** Different reagents ($ZnSO_4$, hydrogen peroxide or GSH/GSSG) were used to optimize FRPcc oxidation. The disulfide crosslinking of dimers was confirmed by SDS-PAGE after pre-treatment of the samples in 20 mM βME and boiling. Note that $ZnSO_4$ and H_2O_2 induced visible sample aggregation, whereas H_2O_2 also affected the electrophoretic behavior of FRP, suggestive of too harsh protein modification. Control corresponds to the untreated sample. **c** Crosslinking of the FRPcc mutant under optimal conditions resulted in crosslinking of >95 % protein. Boiling in sample buffer with 20 mM βME and SDS completely disrupted the dimer. M_W markers (kDa) are shown on the right of (**c**).

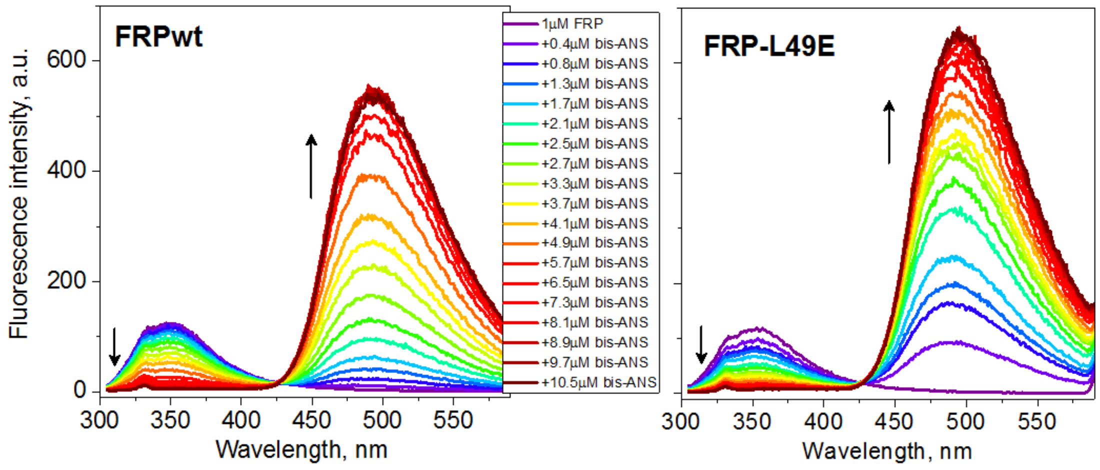

Supplementary Fig. 2 Hydrophobic properties of the FRP variants. Titration of FRPwt and FRP-L49E (1 μM) by bis-ANS (0-10.5 μM) followed by spectral changes in the 305-590 nm region (excitation at 297 nm) at 20 °C. Arrows indicate the direction of changes in the course of titration. These data were used to plot binding curves presented in Fig. 2d of the main text.

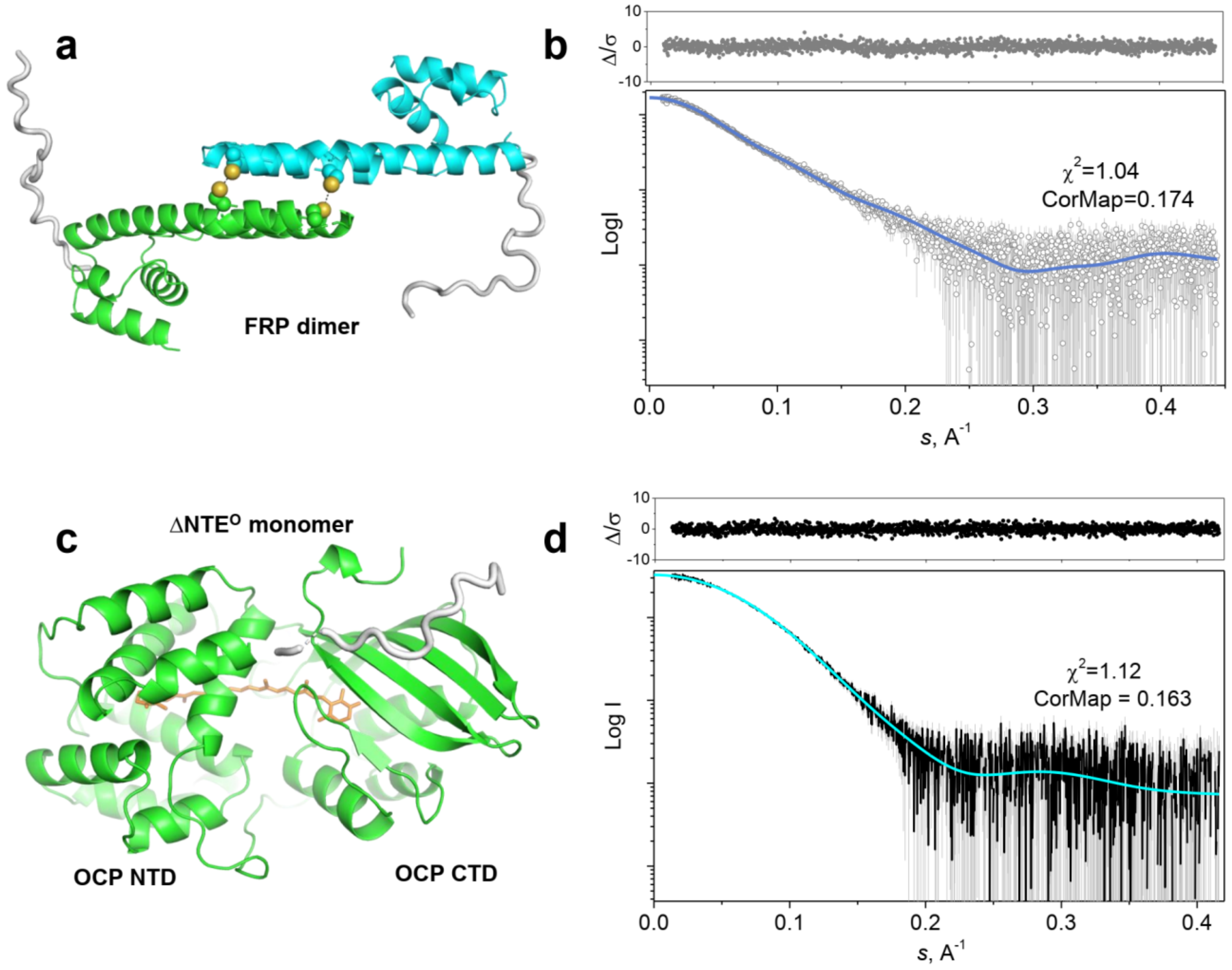

Supplementary Fig. 3 Solution conformation of oxFRPcc and ΔNTE^{O} analyzed by SAXS. The best fitting model of dimeric FRPcc with disulfide bridges fixing the interface and flexible N-terminal tags (**a**) and its fit to the experimental SAXS data corresponding to 1.7 mg ml^{-1} protein concentration on the whole range of scattering vectors (**b**), as well as the corresponding residuals (Δ/σ), are shown. FRP subunits are in cyan and green, white ribbons show flexible N-terminal tags modeled by CORAL (see Methods). Yellow spheres designate the engineered disulfide bridges C33-C43. The protein sample was pre-oxidized by dialysis against GSH/GSSG and was checked to be ~95 % crosslinked according to SDS-PAGE prior to SAXS measurements. **c** Structural model of the ΔNTE^{O} monomer obtained by truncating the crystallographic OCP monomer (PDB ID: 4XB5; residues 1-20 removed) and modeling of 13 missing N-terminal residues (shown by white ribbon) in CORAL[2] to maximize the correspondence to the experimental data. The carotenoid is shown in orange. **d** The fit of the CORAL model to the SAXS data calculated by CRYSOL[3] over the whole range of scattering vectors, as well as the corresponding residuals (Δ/σ).

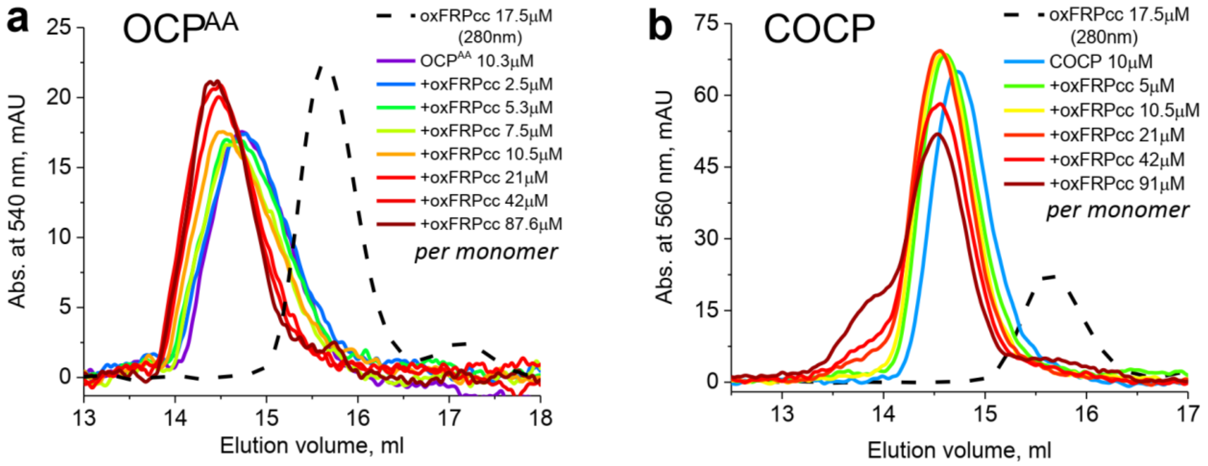

Supplementary Fig. 4 Interaction of the pre-oxidized FRPcc mutant with either OCP^{AA} (**a**) or COCP (**b**). SEC was performed on a Superdex 200 Increase 10/300 column using a fixed (10 µM) concentration of either OCP^{AA} or COCP and varying concentrations of oxFRPcc (indicated per monomer). Flow rate 1.2 ml min^{-1}. The elution profiles were followed by absorbance at the indicated wavelengths.

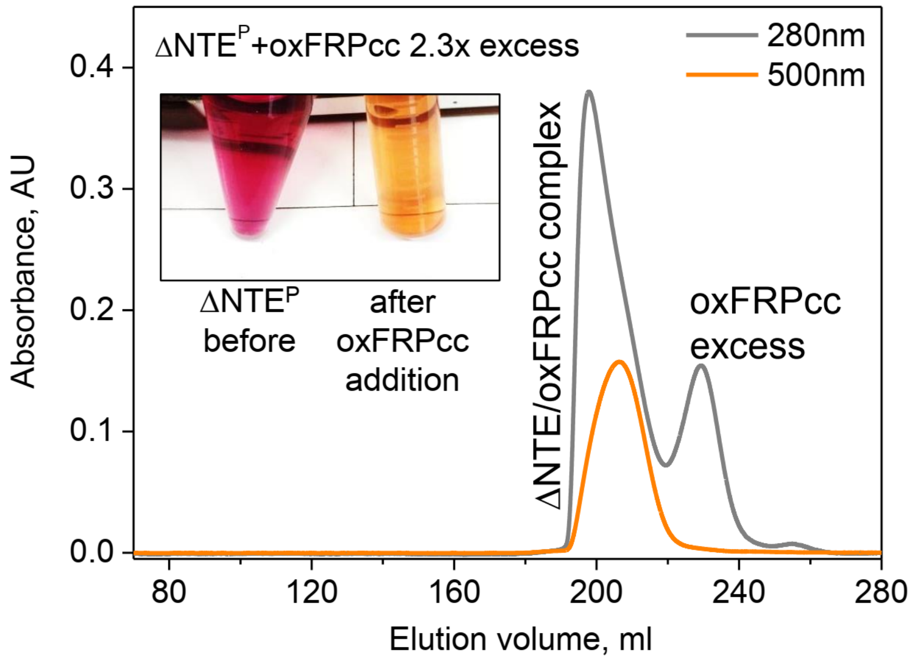

Supplementary Fig. 5 Interaction of ΔNTE^{P} with separated domains and oxFRPcc. ΔNTE^{P} was mixed with oxFRPcc taken in 2.3 molar excess and separated by SEC on a Superdex 200 26/600 column with protein- (grey line) and carotenoid-specific (orange line) detection. Ten min after mixing the proteins, characteristic oranging of the purple OCP form took place, indicating efficient OCP–FRP interaction[4, 5]. Flow rate was 2.6 ml min^{-1}.

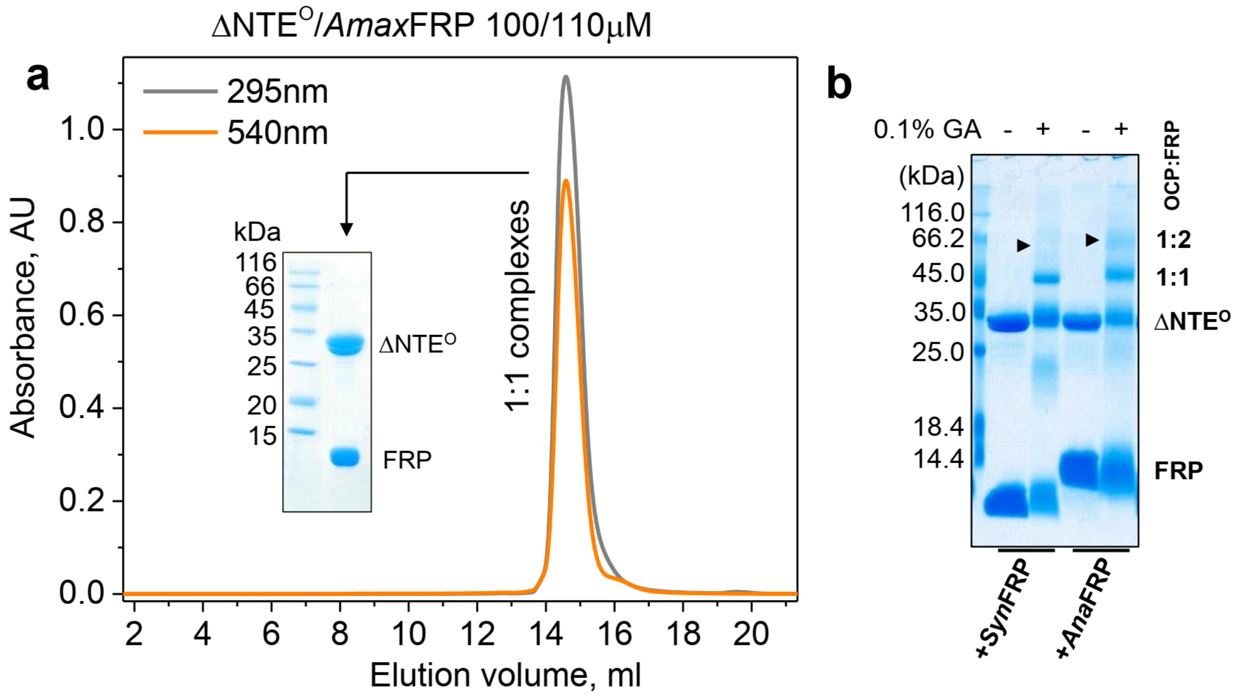

Supplementary Fig. 6 Monomerization of FRP upon its interaction with ΔNTE^O. **a** SEC of the ΔNTE^O mixture with a slight excess of *A. maxima* FRP on a Superdex 200 Increase 10/300 column. The *Arthrospira* homolog of FRP was taken because of its preferential ability to form almost exclusively 1:1 complexes with OCP forms[6]. The profile indeed shows mostly 1:1 interaction with no appreciable excess of ΔNTE^O, which would have been expected for OCP–FRP binding at a 1:2 stoichiometry. The peak fraction contents were analyzed by SDS-PAGE (insert), which confirmed 1:1 stoichiometry[6]. **b** Comparison of the crosslinking of ΔNTE^O with either *Synechocystis* or *Anabaena* FRP containing a different number of potentially crosslinkable residues in the interface. Note that although the efficiency of the 1:2 band crosslinking (indicated by arrowheads) is increased in the case of *Anabaena* FRP, probably, due to the presence of four Lys residues in the dimerization region, the intensity of the 1:1 band even in this case is much larger, implying at least partial FRP monomerization.

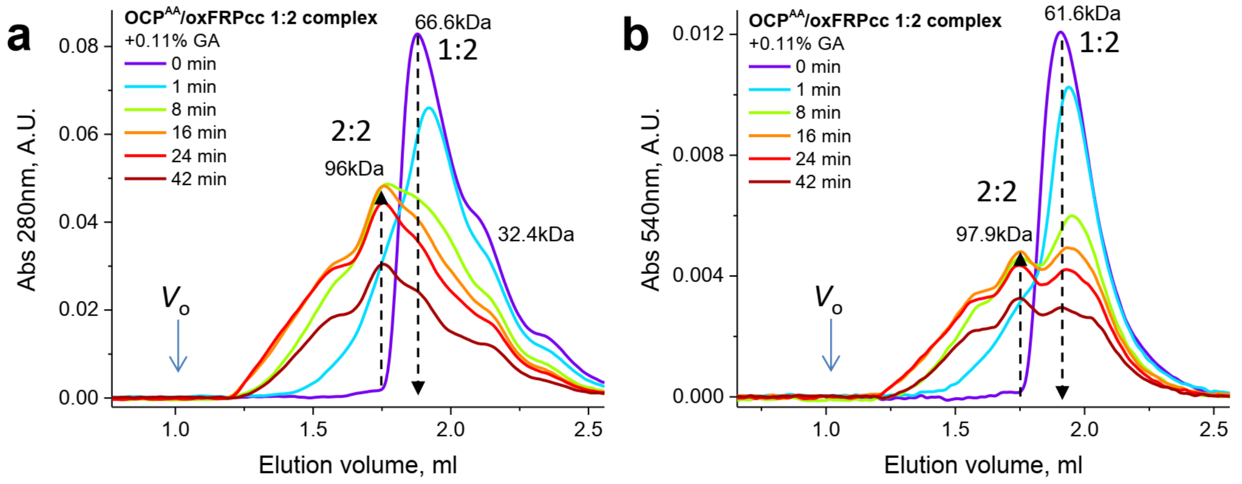

Supplementary Fig. 7 GA crosslinking helps detecting metastable 2:2 OCP–FRP complexes. Kinetics of the crosslinking of the mixture of the analog of the photoactivated OCP form, OCP^{AA}, and oxFRPcc by 0.11 % GA (final concentration) incubated at room temperature as analyzed by SEC on a Superdex 200 Increase 5/150 column (GE Healthcare) at a 0.45 ml min^{-1} flow rate upon loading 30 μl aliquots of the reaction mixture at different time points. The decrease of the peak of the 1:2 complex (60-66 kDa) and a concomitant increase of the peak of the 2:2 complex (96-99 kDa) in the course of titration are marked by arrows. SEC profiles were followed by either protein-specific (**a**) or carotenoid-specific (**b**) absorbance, void volume is indicated (V_o).

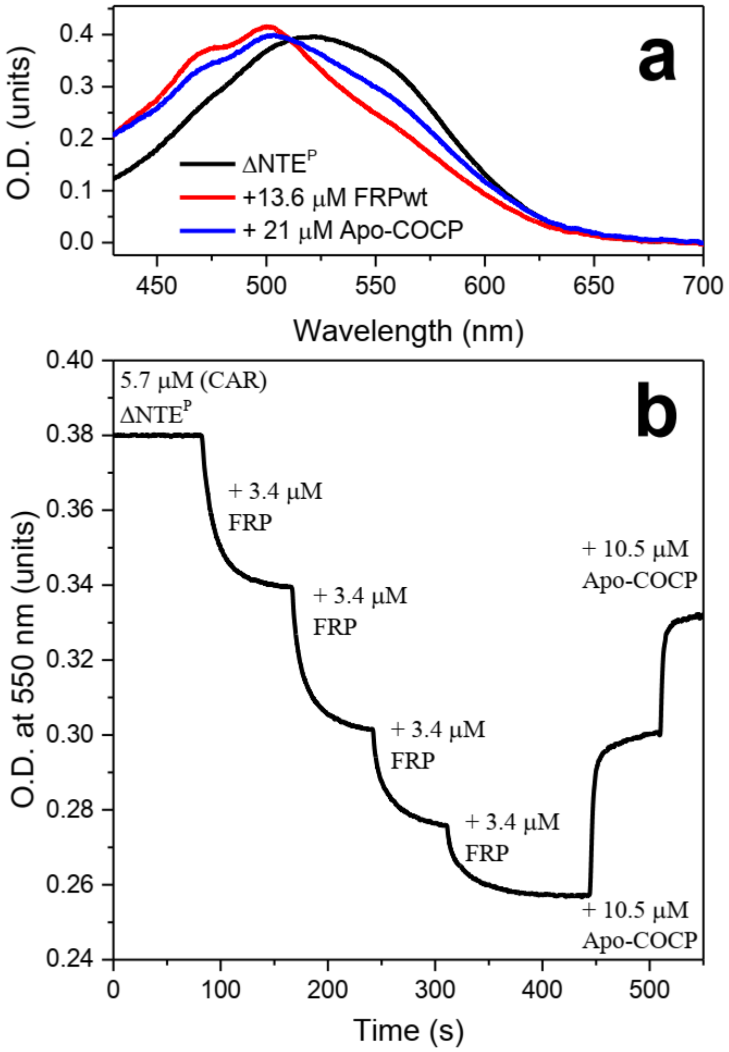

Supplementary Fig. 8 Dynamic nature of ΔNTE^{O}–FRP complexes. The experiment demonstrates that even though different methods show that FRP tightly interacts with its binding site in the CTD of ΔNTE^{O}, it does not stay in contact all the time, probably due to some as yet unknown constrains. To test this hypothesis, we used initially purple ΔNTE^{P} (coordinating canthaxanthin) as a model of OCP with separated domains, which could be united by FRP, providing a possibility for the carotenoid to form bonds with CTD residues and thus change its color to orange (**a**). The idea was to test if this oranging is reversible due to the ability of FRP to spontaneously detach from ΔNTE. We found that upon addition of Apo-COCP (CTDs) to the solution containing orange ΔNTE^{P}–FRP complexes, its color changes to purple, as monitored by changes of O.D. at 550 nm (**b**), indicating redistribution of FRPs between Apo-COCP and ΔNTE^{P}. Such redistribution is only possible, if the ΔNTE–FRP complex is metastable and dissociating from time to time. Experiments were conducted at 30 °C and constant stirring.

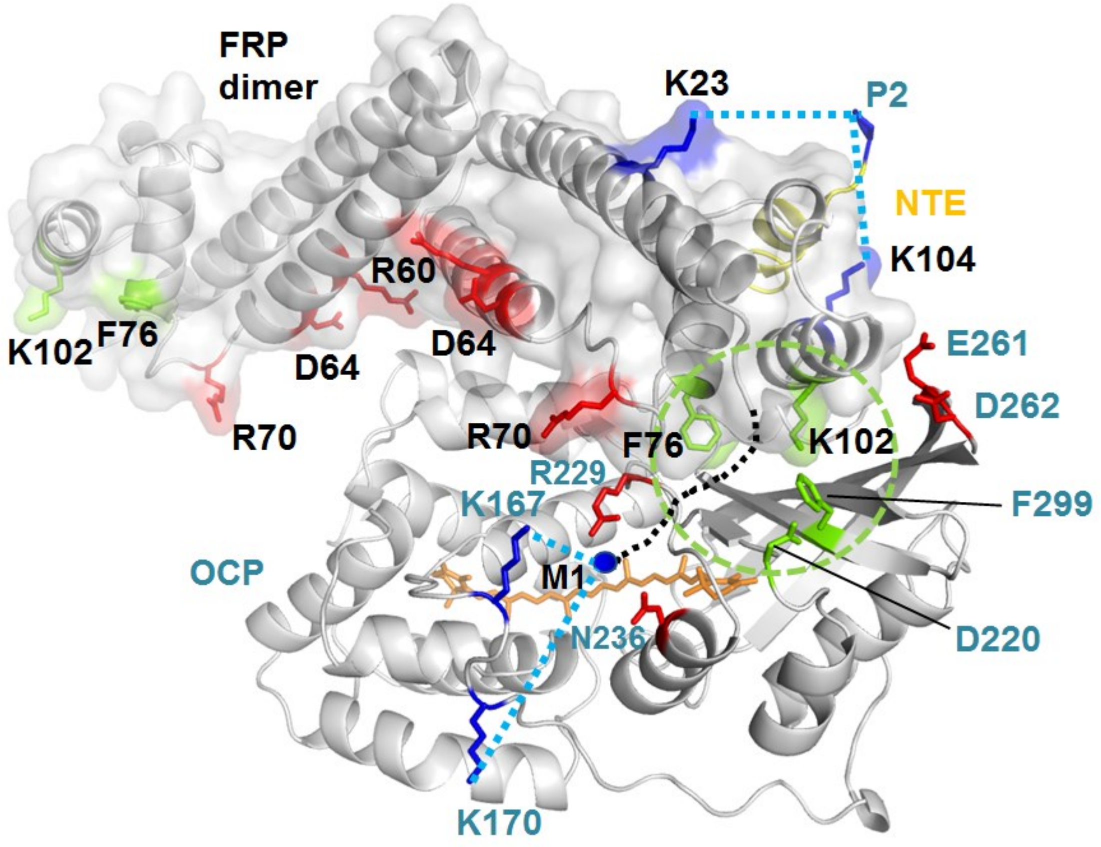

Supplementary Fig. 9 Structural model of the transient 1:2 OCP–FRP complex. OCP and FRP are shown by gray cartoon, FRP dimer is also shown by semitransparent surface. The carotenoid is shown by orange sticks, NTE (yellow) is drawn detached and shifted from its binding site to allow for FRP binding. Residues F76, K102 of FRP and D220, F299 of OCP, replacement of which severely affected OCP–FRP binding[7, 8], are shown by green sticks (also encircled by a green dashed line); whereas residues R229, N236, E261, D262 of OCP and residues R60, D64, R70 of FRP, replacement of which did not severely affect OCP–FRP binding[7, 8, 9, 10], are shown as red sticks. Notably, several observations indicate that mutations of OCP or FRP can hamper FRP R-O converting activity, but not binding to OCP, which agrees with the fact that the productive FRP binding is multisite. Besides the main binding site, transient interactions with the OCP-NTD are plausible and may involve residues (e.g., of R60) from the concave side of the FRP dimer. The reported intermolecular crosslinks[7] were mapped by showing the crosslinkable residues by blue sticks and the shortest distances (≤15 Å) within the experimentally validated pairs (the spacer length was 11.4 Å[7]) as light blue dashed lines. The crosslinks between either K167 or K170 of OCP and M1 of FRP are possible due to the expected flexibility of the N-terminal tail of FRP (residues 1-7 shown by a black dotted line on the proximal FRP subunit), which also explains the detected crosslinking of P2 of OCP and M1 of FRP[7]. The only crosslink that cannot be mapped on our model built for ΔNTE^{O} and formed between K249 of OCP and K23 of FRP[7] likely corresponds to the intermediary binding of FRP to the NTE-containing OCP^{O} utilized in a previous study. The reported increase in solvent accessibility of the D54 residue of FRP upon its interaction with OCP^{R} [7] agrees well with the splitting of the 2:2 complexes into 1:1 subcomplexes and disruption of the FRP interface.

Supplementary Note 1 Modeling of the dissociative mechanism of FRP binding to OCP

Compared to oxFRPcc, FRPwt is capable of monomerization upon OCP binding. Given the fact that simultaneous occupation of two OCP binding sites on a single FRP dimer is hardly possible in vitro due to the steric clash between the OCP molecules, it can be assumed that the cross-linked oxFRPcc can bind only one OCP molecule. In the case of FRPwt, the existence of dissociation principally enables the use of both sites and formation of complexes between OCP and FRP monomers, as observed previously[4]. Hence, as a general rule, FRPwt should have a higher affinity for OCP compared to oxFRPcc: while the first stage of binding (OCP+FRP dimer) is similar for both proteins, in FRPwt the second binding stage is present. Figure 5b (see main text) depicts the OCP binding curves for oxFRPcc and FRPwt obtained in SEC experiments, which yield exactly similar binding affinities (K_d ≈ 0.2 μM). This fact is indicative of the similarity of the first binding stage (1) in dissociation-prone and cross-linked proteins, as well of the negligible impact of other binding stages in the case of FRPwt in the selected range of OCP concentrations (2÷5 μM). To investigate the corresponding processes in more detail, we performed numerical modeling of the binding processes in the OCP–FRP system based on the understanding of the molecular basis of their interaction.

Generally, two schemes of binding between FRPwt and OCP are possible, as shown in Supplementary Figure 10.

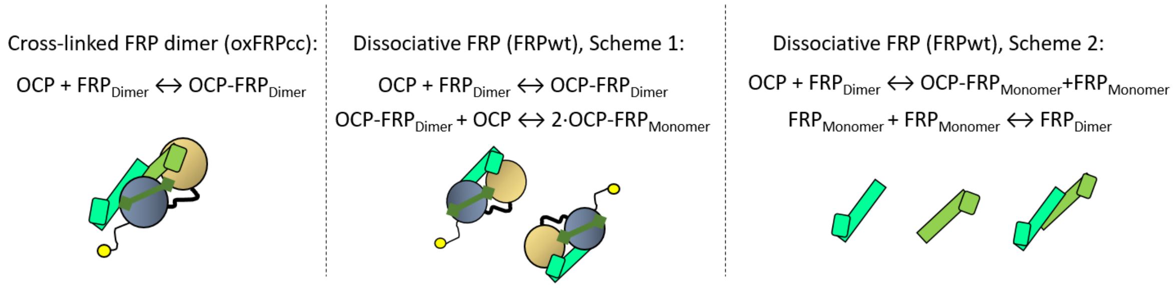

Supplementary Figure 10. Possible schemes of the OCP interaction with FRP.

In the first scheme, monomerization of FRP occurs upon the attempt of binding of the second OCP to the complex of OCP and the FRP dimer. This leads to destabilization of the dimer and consequent formation of OCP complexes with FRP monomers, which are readily observed in the experiments. In the second scheme, binding of a single OCP molecule results in FRP monomerization and formation of free FRP monomers, which consequently form FRP dimers. Obviously, in both of these models, all binding sites on FRP (i.e. two sites per each dimer) can be potentially occupied by OCP. As the main conclusions are similar for the schemes 1 and 2, we will address the case of scheme 1 (Supplementary Figure 11).

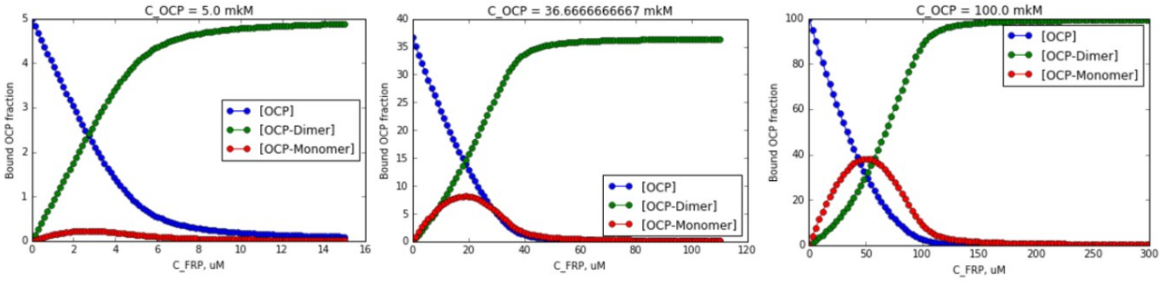

Supplementary Figure 11. The results of modeling of the Scheme 1 for different OCP concentrations. The affinity of FRP dimer for OCP was taken from the experiment (K_d = 0.2 μM), and the efficiency of the second binding stage was chosen 50-fold lower.

As can be seen in Supplementary Figure 11, the formation of OCP complexes with FRP monomers becomes more pronounced at higher OCP concentrations. While for an excess of FRP all OCP is bound to dimers (i.e. there is no necessity in using the second site, and the second binding stage is ineffective), at $C_{FRP} \sim C_{OCP}/2$ the concentration of dimers is at maximum. In other words, at high OCP concentrations, FRP monomerization helps to effectively enhance the binding capacity.

Supplementary references

1. Franke D*, et al.* ATSAS 2.8: a comprehensive data analysis suite for small-angle scattering from macromolecular solutions. *J Appl Crystallogr* **50**, 1212-1225 (2017).

2. Petoukhov MV*, et al.* New developments in the ATSAS program package for small-angle scattering data analysis. *J Appl Cryst* **45**, 342-350 (2012).

3. Svergun DI, Barberato C, Koch MHJ. CRYSOL - a Program to Evaluate X-ray Solution Scattering of Biological Macromolecules from Atomic Coordinates. *J Appl Cryst* **28**, 768-773 (1995).

4. Sluchanko NN, Klementiev KE, Shirshin EA, Tsoraev GV, Friedrich T, Maksimov EG. The purple Trp288Ala mutant of Synechocystis OCP persistently quenches phycobilisome fluorescence and tightly interacts with FRP. *Biochim Biophys Acta* **1858**, 1-11 (2017).

5. Sluchanko NN, Slonimskiy YB, Moldenhauer M, Friedrich T, Maksimov EG. Deletion of the short N-terminal extension in OCP reveals the main site for FRP binding. *FEBS Lett* **591**, 1667-1676 (2017).

6. Slonimskiy YB*, et al.* Functional interaction of low-homology FRPs from different cyanobacteria with Synechocystis OCP. *Biochim Biophys Acta* **1859**, 382-393 (2018).

7. Lu Y*, et al.* A Molecular Mechanism for Nonphotochemical Quenching in Cyanobacteria. *Biochemistry* **56**, 2812-2823 (2017).

8. Thurotte A*, et al.* The cyanobacterial Fluorescence Recovery Protein has two distinct activities: Orange Carotenoid Protein amino acids involved in FRP interaction. *Biochim Biophys Acta* **1858**, 308-317 (2017).

9. Bao H*, et al.* Additional families of orange carotenoid proteins in the photoprotective system of cyanobacteria. *Nature plants* **3**, 17089 (2017).

10. Sutter M*, et al.* Crystal structure of the FRP and identification of the active site for modulation of OCP-mediated photoprotection in cyanobacteria. *Proc Natl Acad Sci U S A* **110**, 10022-10027 (2013).
